# Skin Diseases, Their Pathology Diagnosis and Treatment

**Published:** 1873-09

**Authors:** 


*Skin Diseases. Their Pathology, Diagnosis, and Treatment.* By Tilbury Fox, M. D., London. Second American from Third London Edition, Re-written and enlarged. New York: Wm. Wood & Co., 1873. Buffalo: H. H. Otis.

Since the publication of the first American Edition of this work, it has largely increased in favor among American practitioners and received the highest commendation from all who have had the pleasure of reading it. Its many admirers will be pleased therefore to learn that a new edition has been published under the editorship of Dr. Henry, who had the honor of introducing the former edition to the attention of American Dermatologists. The present edition is so much improved and enlarged over the former one however that its recognition would be almost a matter of difficulty.

Much has been added that is entirely new, and the contents of the first edition have undergone a complete revisal, many new thoughts having been added and obsolete ones expunged. A large number of illustrations have been introduced into this edition, taken in a large measure from the works of German authors. Dr. Fox has not hesitated to take advantage of the labors of other dermatologists both Continental and American, and has in every instance given them credit for whatever he has borrowed. Each disease is treated of in sufficient fullness to be clearly understood by the intelligent reader, and the present edition of Dr. Fox's work may be safely said to be the best English treatise on skin diseases extant.

